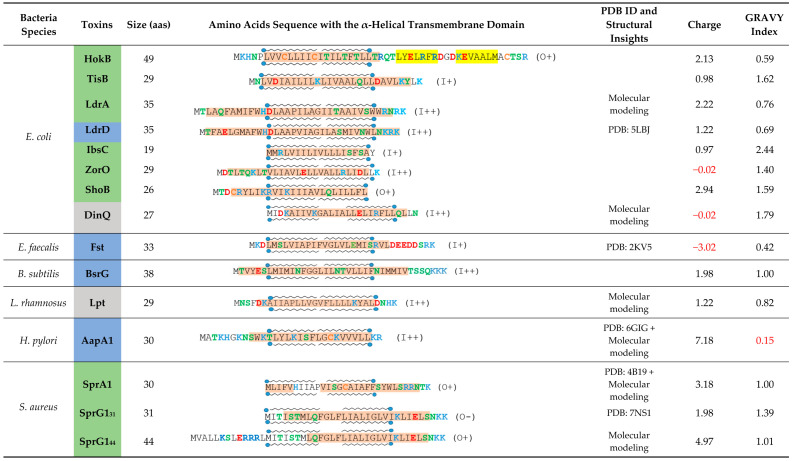# Correction: Nonin-Lecomte et al. Bacterial Type I Toxins: Folding and Membrane Interactions. *Toxins* 2021, *13*, 490

**DOI:** 10.3390/toxins13120878

**Published:** 2021-12-09

**Authors:** Sylvie Nonin-Lecomte, Laurence Fermon, Brice Felden, Marie-Laure Pinel-Marie

**Affiliations:** 1 CiTCoM, CNRS, UMR 8038, Université de Paris, 93526 Paris, France; sylvie.nonin@u-paris.fr; 2 BRM (Bacterial Regulatory RNAs and Medicine), Inserm, UMR_S 1230, Université de Rennes 1, 35000 Rennes, France; laurence.fermon@univ-rennes1.fr (L.F.); brice.felden@univ-rennes1.fr (B.F.)

The authors wish to make the following corrections to their paper [1].

In the original publication, there was a mistake in Table 1 as published. While editing Table 1, there has been a row offset for the “PDB ID and Structural Insights” column. Moreover, the final lysine residue was missing on the BsrG sequence. Consequently, the size, the charge and the GRAVY index have been modified. The corrected Table 1 appears below. The authors apologize for any inconvenience caused and state that the scientific conclusions are unaffected. The original publication has also been updated.

## Figures and Tables

**Table 1:** Overview of membrane-associated type I toxins from toxin-antitoxin systems for which insights into their mechanism of action have been published. Predicted or experimentally determined (according to respective PDB file) α-helix are highlighted in orange and β-sheet in yellow. When the structure has not been experimentally determined, α-helix have been predicted with Jpred4 (http://www.compbio.dundee.ac.uk/jpred/index.html, accessed on 5 April 2021) [27]. Transmembrane domains are delimited by lipid representation surrounding each sequence and have been predicted with TMPRED (https://embnet.vital-it.ch/software/TMPRED_form.html, accessed on 5 April 2021). Boxes colored in blue correspond to the toxins inducing morphological changes as a primary detected effect, the green one for toxins inducing membrane perturbations as a primary detected effect and the grey one is for toxins with dual effects. Polar amino acids are shown in green, negatively charged amino acids in red and positively charged amino acids in blue. Cysteins are shown in orange. The charge and the hydrophobicity index (based on Kyte-Doolittle scale) have been calculated thanks to the R package «Peptides» [28,29]. Unexpected results like low hydrophobicity or global negative charge have been written in red.