# Supplementary material for: Bacterial Type I Toxins: Folding and Membrane Interactions
Source: Toxins (Basel). 2021 Jul 14;13(7):490. doi: 10.3390/toxins13070490 (PMC8309996; doi:10.3390/toxins13070490)
Supplement: Supplementary file 1 [file toxins-13-00490-s001.zip › toxins-1271363-supplementary.pdf]

Supplementary Materials: Bacterial Type I Toxins: Folding and Membrane Interactions

Sylvie Nonin-Lecomte, Laurence Fermon, Brice Felden and Marie-Laure Pinel-Marie

S1. NMR sample preparation and experiments

The NMR samples consist of either 0.3mM $SprG1_{31}$ or $SprG1_{44}$ peptide solutions supplemented with EDTA 0.01mM in H_2O:*d8*-Isopropanol 50%:50% v/v buffer at pH 4.5. The spectra were recorded on a 600MHz Bruker Avance III spectrometer equipped with a TCI cryo-probe and Z-axis gradient, using Shigemi 5mm tubes plugged and sealed to avoid isopropanol evaporation. Two-dimensional ^1H-^{1H} TOCSY (80ms mixing time) and NOESY (80ms, 150ms, and 300 ms mixing times) were acquired at different temperatures (298, 303, and 313K). Raw data were processed with TOPSPIN 3.5.7 (Bruker Biospin). Peak peaking, chemical shift assignments, peak intensities, and volume measurements and distance conversions were carried out with CcpNmr Analysis 2.4.2 [1]. The distance restraints list was implemented using ARIA 2.3.2 software [2] coupled to CNS 1.2.1 [3] and Analysis 713 NOE distance restraints were derived (182 sequential, 162 medium range, 48 long range i-i+4). Chemical shifts have been deposited to BMRB (accession number 50767).

S2. Structure determination

Simulated annealings, refinements, and minimizations were carried out using XPLOR-NIH 3.1 software [4] and the distance restraints list generated by CcpNmr Analysis 2000 structures were calculated in a vacuum starting from a random extended peptide. The 500 structures of lowest energies with no NOE violations over 0.3 Å were refined in an implicit solvent using a nonbonded EEFx [5] and statistical torsion angle tDB [6] potentials and were minimized. The ten structures of lowest energies with no NOE violations above 0.25 Å and satisfying Molprobitiy [7] scores below 2.25 were selected for RMSD computations. The coordinates of the five best structures have been deposited in the PDB data bank (7NS1).

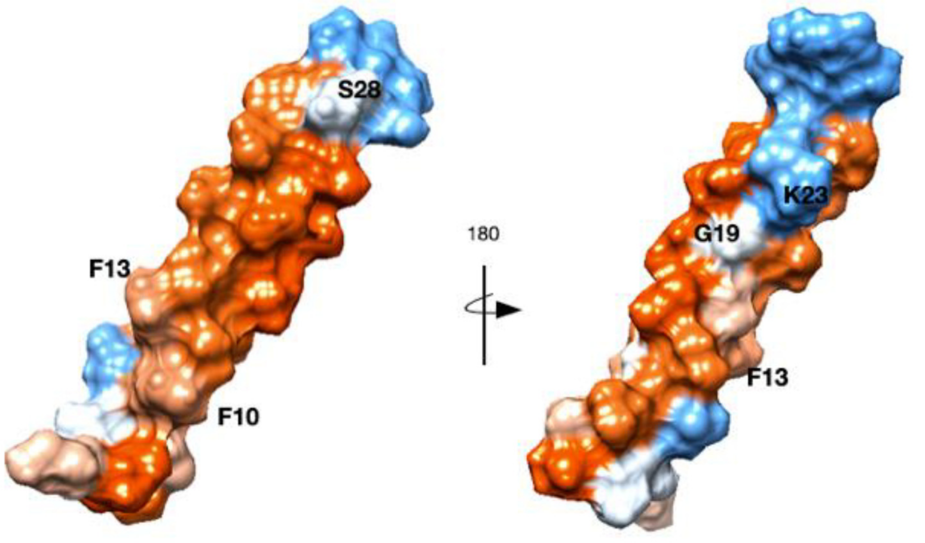

Figure S1. $SprG1_{31}$ peptide: hydrophobicity surface.

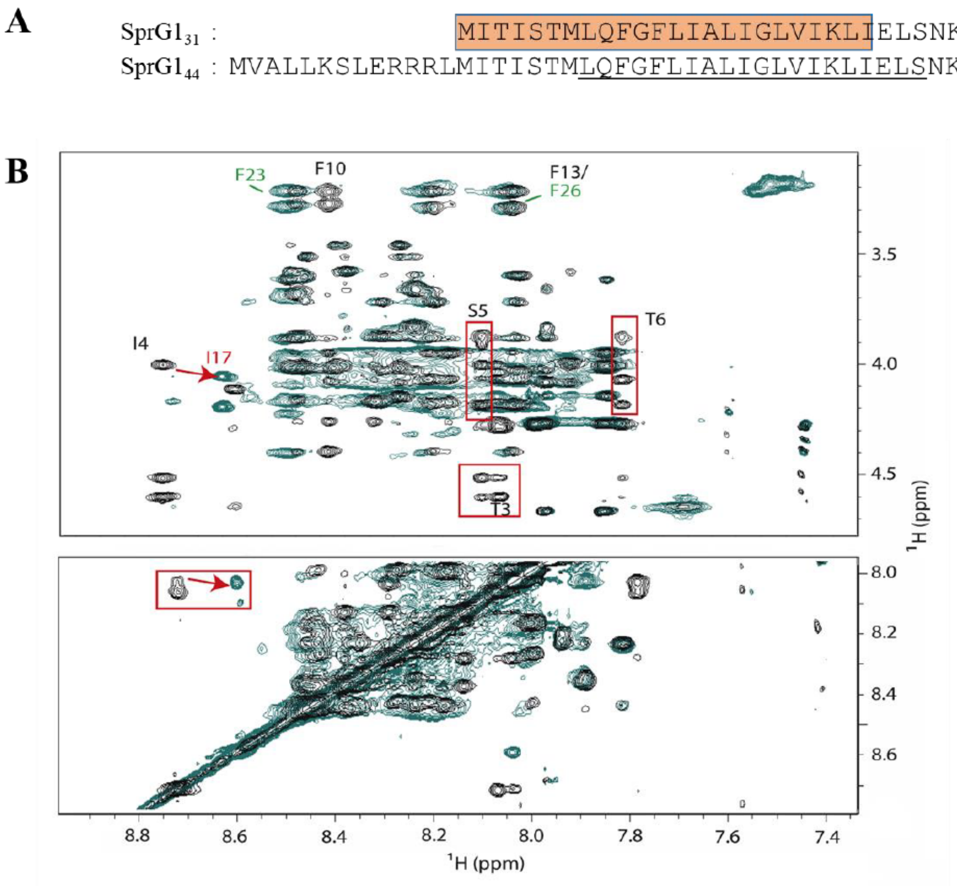

Figure S2: (**A**)**:** Alignment of SprG1$_{31}$ and SprG1$_{44}$ sequences. SprG1$_{31}$ α-helix is highlighted in orange. The resonances of the SprG1$_{44}$ underlined residues display similar NOESY cross-peaks as their homologs in SprG$_{31}$. (**B**): Superposition of the 300ms NOESY spectra of SprG1$_{31}$ (black) and SprG1$_{31}$ (green) peptides recorded at 313K and pH 4.5 in 50% v/v *d8*-isopropanol. Many amino acids of both peptides display good peak superposition, including F23(F10) and F26(F13). SprG1$_{44}$ L21-S41 connectivities (underlined residues in panel A) are similar to SprG1$_{31}$ L8-S28, showing that both segments adopt similar 3D structures. Many intra and inter residue cross-peaks of the I15-M20 (I4-M7) segment are either enlarged or missing in the region (red squares), showing that this part of the peptide is very dynamic and destabilized by the extra M1-L13N-ter segment.

References

1. Vranken, W.F.; Boucher, W.; Stevens, T.J.; Fogh, R.H.; Pajon, A.; Llinas, M.; Ulrich, E.L.; Markley, J.L.; Ionides, J.; Laue, E.D. The CCPN Data Model for NMR Spectroscopy: Development of a Software Pipeline. *Proteins* **2005**, *59*, 687–696, doi:10.1002/prot.20449.
2. Rieping, W.; Habeck, M.; Bardiaux, B.; Bernard, A.; Malliavin, T.E.; Nilges, M. ARIA2: Automated NOE Assignment and Data Integration in NMR Structure Calculation. *Bioinformatics* **2007**, *23*, 381–382, doi:10.1093/bioinformatics/btl589.
3. Brünger, A.T.; Adams, P.D.; Clore, G.M.; DeLano, W.L.; Gros, P.; Grosse-Kunstleve, R.W.; Jiang, J.S.; Kuszewski, J.; Nilges, M.; Pannu, N.S.; et al. Crystallography & NMR System: A New Software Suite for Macromolecular Structure Determination. *Acta Crystallogr D Biol Crystallogr* **1998**, *54*, 905–921, doi:10.1107/S0907444998003254.
4. Schwieters, C.; Kuszewski, J.; Mariusclore, G. Using Xplor–NIH for NMR Molecular Structure Determination. *Progress in Nuclear Magnetic Resonance Spectroscopy* **2006**, *48*, 47–62, doi:10.1016/j.pnmrs.2005.10.001.

5. Tian, Y.; Schwieters, C.D.; Opella, S.J.; Marassi, F.M. A Practical Implicit Solvent Potential for NMR Structure Calculation. *Journal of Magnetic Resonance* **2014**, *243*, 54–64, doi:10.1016/j.jmr.2014.03.011.
6. Bermejo, G.A.; Clore, G.M.; Schwieters, C.D. Smooth Statistical Torsion Angle Potential Derived from a Large Conformational Database via Adaptive Kernel Density Estimation Improves the Quality of NMR Protein Structures: Smooth Statistical Torsion Angle Potential. Protein Science 2012, 21, 1824–1836, doi:10.1002/pro.2163.
7. Chen, V.B.; Arendall, W.B.; Headd, J.J.; Keedy, D.A.; Immormino, R.M.; Kapral, G.J.; Murray, L.W.; Richardson, J.S.; Richardson, D.C. MolProbity : All-Atom Structure Validation for Macromolecular Crystallography. Acta Crystallogr D Biol Crystallogr 2010, 66, 12–21, doi:10.1107/S0907444909042073.
